# *Babesia duncani* Pyruvate Kinase Inhibitor Screening and Identification of Key Active Amino Acid Residues

**DOI:** 10.3390/microorganisms12061141

**Published:** 2024-06-04

**Authors:** Fangjie Li, Pengfei Zhao, Sen Wang, Wanxin Luo, Yingjun Xia, Dongfang Li, Lan He, Junlong Zhao

**Affiliations:** 1 State Key Laboratory of Agricultural Microbiology, College of Veterinary Medicine, Huazhong Agricultural University, Wuhan 430070, China; lfj@webmail.hzau.edu.cn (F.L.); pengfeizhao0513@163.com (P.Z.); senwang@webmail.hzau.edu.cn (S.W.); luowanxin@webmail.hzau.edu.cn (W.L.); 2018302120125@webmail.hzau.edu.cn (Y.X.); dongfang0216@webmail.hzau.edu.cn (D.L.); helan@mail.hzau.edu.cn (L.H.); 2 Key Laboratory of Preventive Veterinary Medicine in Hubei Province, The Cooperative Innovation Center for Sustainable Pig Production, Wuhan 430070, China; 3 Key Laboratory of Development of Veterinary Diagnostic Products, Ministry of Agriculture of the People’s Republic of China, Wuhan 430070, China

**Keywords:** *Babesia duncani*, pyruvate kinase I, inhibitors, key amino acids, inhibition assay

## Abstract

*Babesia duncani* (*B. duncani*), a protozoan parasite prevalent in North America, is a significant threat for human health. Given the regulatory role of pyruvate kinase I (PyK I) in glycolytic metabolism flux and ATP generation, PyK I has been considered the target for drug intervention for a long time. In this study, *B. duncani* PyK I (BdPyK I) was successfully cloned, expressed, and purified. Polyclonal antibodies were confirmed to recognize the native BdPyK I protein (56 kDa) using Western blotting. AlphaFold software predicted the three-dimensional structure of BdPyK I, and molecular docking with small molecules was conducted to identify potential binding sites of inhibitor on BdPyK I. Moreover, inhibitory effects of six inhibitors (tannic acid, apigenin, shikonin, PKM2 inhibitor, rosiglitazone, and pioglitazone) on BdPyK I were examined under the optimal enzymatic conditions of 3 mM PEP and 3 mM ADP, and significant activity reduction was found. Enzyme kinetics and growth inhibition assays further confirmed the reliability of these inhibitors, with PKM2 inhibitor, tannic acid, and apigenin exhibiting the highest selectivity index as specific inhibitors for *B. duncani*. Subsequently, key amino acid residues were mutated in both BdPyK I and Homo sapiens pyruvate kinase I (HPyK I), and two differential amino acid residues (isoleucine and phenylalanine) were identified between HPyK I and BdPyK I through PyK activity detection experiments. These findings lay foundation for understanding the role of PyK I in the growth and development of *B. duncani*, providing insights for babesiosis prevention and drug development.

## 1. Introduction

Babesiosis, an emerging tick-borne disease, is caused by intracellular protozoan parasites from the *Babesia* genus [1]. Various species capable of infecting humans have been identified, including *Babesia microti*, *Babesia divergens*, *Babesia duncani*, and *Babesia bigemina* [1,2,3]. The clinical spectrum of babesiosis varies widely, from mild symptoms such as fever and headache to severe complications like multi-organ failure, potentially leading to mortality [4,5]. These species exhibit diversity in transmission modes, clinical manifestations, and pathological characteristics, posing a significant threat to human health [3,6]. 

Babesiosis is prevalent among mammals, significantly impacting livestock, wildlife, and pets, and causes considerable economic losses to the agriculture industries. *B. duncani*, initially identified in 1991 from a clinical specimen of a patient without typical risk factors such as advanced age, splenectomy, and immunodeficiency [7], is primarily transmitted by the hard tick *Dermacentor albipictus* [8]. Recent investigations using continuous in vitro culture of human erythrocytic parasites have demonstrated the low sensitivity of *B. duncani* to atovaquone, quinine, clindamycin, and azithromycin, with a resurgence observed three days post-exposure to high concentrations of atovaquone or azithromycin [9,10]. Current treatment protocols encounter obstacles like drug resistance and side effects, underscoring the urgent necessity for exploring novel drug targets and developing innovative therapeutics to address these issues.

Drug discovery efforts often revolve around identifying unique metabolic pathways or vital enzymes within these pathways as potential targets. Among these, the glycolytic pathway has garnered attention for drug development due to its critical role in ATP production, a molecule vital for most metabolic synthesis pathways in parasitic life cycles [11]. Previous studies suggested that parasite infection notably elevates pyruvate kinase (PyK) levels in hosts [12,13]. PyK is a key regulatory factor governing metabolic flux in glycolysis and ATP production. However, the conserved nature of the PyK active site and the central significance of glycolysis across all organisms pose challenges for developing selective inhibitors targeting PyK active site. PyKs are almost subject to allosteric regulation by various effectors, suggesting that focusing on the distinct active sites of PyKs is an efficacious therapeutic strategy against *Babesia* species reliant on glycolysis for energy production [14,15]. Improving selectivity in inhibiting PyK can be a promising method for developing novel treatments against *Babesia* infection, addressing the limitations by the conserved nature of the glycolytic pathway.

The apicomplexan parasite’s PyK is classified into two types: PyK I, localized in the cytoplasm, and PyK II, localized in the apicoplast. Studies on *Toxoplasma gondii* have indicated that the deletion of the PyK II gene does not significantly affect the parasite’s growth and virulence [16]. However, targeted deletion of PyK I leads to comprehensive changes in carbon metabolism, accumulation of branched starch, and reduced cellular ATP, resulting in severe growth impairments [17]. Because *B. duncani* possessed PyK I and PyK II, PyK I may have a crucial role in the sugar metabolism of *B. duncani*. Therefore, this study focuses on investigating BdPyK I. In mammals, PyK exists in three distinct forms: PKL expressed in the liver, kidney, and intestines, PKR expressed in red blood cells, and pyruvate kinase M1 (PKM1) and pyruvate kinase M2 (PKM2) expressed in tissues characterized by high metabolic rates and rapid cell proliferation [18]. PKM2 plays a crucial role in metabolism and growth of tumor in humans, corresponding to HPyK I as well [19]. PKM2 is notably upregulated in most tumors, and its inactive state contributes to tumor metabolism. Moreover, PKM2 can be transformed into dimeric structures from tetrameric forms and is transmitted to the nucleus through post-translational modifications [20,21], which reveal the underlying mechanisms of PKM2 activity regulation. Exploration of the mechanism of PKM2 activity regulation holds important significance for cancer research and therapy targets in tumors.

A variety of compounds have demonstrated effective inhibition of PK activity, including thiazolidinediones, dye derivatives, flavonoids, and naphthoquinone derivatives [22]. Rosiglitazone, a thiazolidinedione drug commonly prescribed for alleviating symptoms of type 2 diabetes, also exhibits neuroprotective features and promises in treating central nervous system diseases such as Parkinson’s, Alzheimer’s, and strokes [23]. Similarly, pioglitazone has demonstrated effects on depression [24]. Subsequent research has unveiled that rosiglitazone can alleviate depressive symptoms by regulating brain glucose metabolism and the AMPK/mTOR signaling pathway [25]. PKM2 inhibitor, a novel naphthoquinone derivative and a specific inhibitor of the tumor cell-specific M2 isoform of PyK, offers significant protection against endotoxin shock in mice and contributes to the attenuation of spontaneous lupus progression [26,27]. Shikonin, the active chemical component derived from lithospermum erythrorhizon, has been identified as a novel tumor PKM2 inhibitor and prevents the conformational transition of tetramer to dimer to inhibit cancer cell glycolysis [28]. Furthermore, apigenin, an aromatic compound found in various fruits and plants, selectively binds to the K433 site of PKM2, blocking PKM2-mediated aerobic glycolysis and exerting anti-colorectal cancer effects in vitro and in vivo [29]. Tannic acid, a natural polyphenolic acid present in grapes and green tea, exhibits potent antioxidant and anticancer properties. Tannic acid directly binds to lysine 433, triggering dissociation of PKM2 tetramers and further impeding PKM2 metabolic activity, thus inhibiting the proliferation of colorectal cancer cells [29]. These findings reveal the potential roles of various compounds in targeting PKM2 activity, presenting promising methods for the development of novel therapeutics against cancer and other diseases.

Currently, few studies report inhibitors targeting BdPyK I. Given the pivotal role of PyK in parasite metabolism and growth, the development of specific inhibitors against this enzyme is essential for the prevention and treatment of babesiosis caused by *B. duncani*. This study aims to predict and analyze the differences between BdPyK I and the mammalian tumor-affecting factor HPyK I (PKM2). This study screened several small-molecule compounds via protein structure binding and enzyme kinetics and identified specific inhibitors of BdPyK I through growth inhibition experiments in vitro. These findings not only enhance comprehension of the critical active amino acid sites between BdPyK I and HPyK I, but also provide valuable insights for the development of specific inhibitors of PyK and novel strategies for the treatment of associated diseases and drug development. 

## 2. Materials and Methods

### 2.1. Preparation of Recombinant Plasmid

Primers (BdPyK I-F: 5′-GTTATCCCTGGGCAAC-GCCTTG-3′, BdPyK I-R: 5′-GTTATCCCTGGGCAACGCCTTG-3′) were designed from Tianyi Huiyuan Biological Technology (Biological Technology, Wuhan, China) based on the PyK I nucleotide sequence (GenBank accession: KAK2194634.1), which was selected from the *B. duncani* genome in NCBI database (Table S1). BdPyK I was amplified from both *B. duncani* cDNA and gDNA. PCR reaction consisted of an initial denaturation step at 95 °C for 5 min, followed by 35 cycles of denaturation at 95 °C for 30 s, annealing at 55 °C for 30 s, extension at 72 °C for 1 min, and a final extension step at 72 °C for 5 min. PCR productions were subjected to electrophoresis on a 2% agarose gel stained with ethidium bromide, followed by purification using the EasyPure^®^ Quick Gel Extraction Kit (Invitrogen, Carlsbad, CA, USA). The purified BdPyK I gene fragments were ligated into the pGEX-6p-1 vector (Takara Biotechnology, Beijing, China) using the ClonExpress II One Step Cloning Kit (Vazyme Biotech, Nanjing, China). Sequencing of the BdPyK I gene was performed using sequencing primers specific to the pGEX-6p-1 vector (Solarbio, Beijing, China).

HPyK I (GenBank accession: NM_002654.6) was amplified using primers (HPyK I-F: 5′-ATGTCGAAGCCCATAGTGAAGC-3′) and HPyK I-R (5′-TCACGGCACAGGAACAAACG-3′) from HeLa cells (Beyotime Biotechnology, Shanghai, China). The Q5 Site-Directed Mutagenesis Kit (NEB, Beijing, China) was utilized to obtain mutant BdPyK I and HPyK I plasmids (Table S1). Mutations were introduced to convert BdPyK I residues 67A (ARG), 71A (SER), 107A (GLU), 108A (ILE), 109A (ARG), 193A (ASN), 230A (PHE), 256A (LYS), 282A (ASP), and 286A (GLU) to alanine (Ala, CCU, CCC, CCA, CCG). All recombinant plasmid sequences were confirmed by DNA sequencing.

### 2.2. Preparation and Purification of Polyclonal Antibodies against BdPyK I

The constructed plasmids were transformed into *E. coli* BL21 (*Escherichia coli*, TransGEN, Beijing, China) and cultured at 37 °C. Induction was performed with 1 mM isopropyl β-D-1-thiogalactopyranoside (IPTG) at 18 °C for approximately 12 h. After induction, BL21 cells were collected by centrifugation at 7000 rpm for 10 min using a high-speed refrigerated centrifuge (Hitachi, Tokyo, Japan). Subsequently, the cells were resuspended in 30 mL of PBS binding buffer (8 g NaCl, 0.2 g KCl, 3.63 g Na_2_HPO_4_·12H_2_O, 0.24 g KH_2_PO_4_) and lysed using a high-pressure homogenizer at 1000 bar. Following lysing, the cell lysate was centrifuged at 10,000 rpm for 10 min at 4 °C, and the supernatant was collected and filtered through a 0.45 μm filter. The filtered supernatant was then loaded onto a GSH Purpose 4 Fast Flow column (Sangon Biotech, Shanghai, China) and eluted with 2 mM reduced glutathione. Subsequently, the purified protein was concentrated and stored at 4 °C. The GST tag was cleaved from the purified protein using laboratory-preserved HRV 3C protease, with the addition of 20 μL DTT (1 mM) and 300 μL HRV 3C enzyme (approximately, 1 mg/mL) at 4 °C overnight. The GST-free rBdPyK I was obtained through filtration and then slowly concentrated using dialysis tubing. The protein concentration was determined using the BCA protein assay kit (Beyotime Biotechnology, Shanghai, China), and the purified protein was stored at −80 °C until use.

The Freund’s complete adjuvant (MilliporeSigma, Burlington, MA, USA) was thoroughly mixed with recombinant protein rBdPyK I in appropriate proportions to achieve a final concentration of 500 μg/mL. This mixture was then injected into Japanese Long Ear Rabbits (5 months old, about 2.8 kg) in four separate doses. One week after the fourth immunization, whole blood was collected from the rabbits via cardiac puncture. The collected blood was incubated at 37 °C for 1 h, followed by overnight incubation at 4 °C. Serum was then separated by centrifugation at 600 g for 10 min and stored at −20 °C.

### 2.3. The Hamster Donor Blood and the Culture of Babesia duncani

Blood samples were obtained from hamsters for isolation of donor red blood cells using EDTA K2 solution (with a solution to red blood cell ratio of 1:9, containing 10% EDTA-2K). The blood collection procedure was performed via retro-orbital venipuncture under isoflurane anesthesia. All blood samples were centrifuged at 500× *g* for 10 min, followed by three washes with PSG (at a volume of 5 times that of the blood) to eliminate plasma and leukocytes. Careful removal of the supernatant and leukocytes was performed during each washing step. Subsequently, the washed red blood cells were combined with an equal volume of PSG with additional glucose (20 g glucose/L, final concentration of 200 μg/mL streptomycin and 200 U/mL penicillin) and stored at 4 °C for a maximum of 2 weeks. The *Babesia duncani* WA1 strain (ATCC PRA-302™) was preserved by the State Key Laboratory of Agricultural Microbiology, College of Veterinary Medicine, Huazhong Agricultural University. We conducted in vitro serum-free culture of *B. duncani* using VP-SFM AGTTM (Gibco Life Technologies, Shanghai, China) as the basal medium. Supplementary additives, such as AlbuMaxTM I or CD lipid mixture (both from Gibco Life Technologies, Shanghai, China), were incorporated into the medium. Additionally, extra supplements, including 200 mM L-glutamine (Sigma, Shanghai, China), and 2% antibiotic/antifungal 100× (Corning, Shanghai, China), were introduced. The parasites were cultured under static conditions at 37 °C with a microaerophilic atmosphere composed of 5% CO_2_, 2% O_2_, and 93% N_2_.

### 2.4. Western Blotting Analysis and Indirect Immunofluorescence Assay

To detect native BdPyK I in *B. duncani*, we employed protein imprinting technology. Initially, *B. duncani* lysates were separated via sodium dodecyl sulfate-polyacrylamide gel electrophoresis (SDS-PAGE) with 12% acrylamide and transferred onto polyvinylidene fluoride (PVDF) membranes. Subsequently, the membrane was treated with a 1% (*w*/*v*) solution of bovine serum albumin (BSA, BioFroxx, Hesse, Germany) at room temperature for 1 h to block nonspecific binding. The membrane was subsequently incubated with rabbit anti-rBdPyK I polyclonal serum (diluted 1:200) at room temperature for 2 h. As controls, lysates from uninfected *B. duncani* red blood cells reacted with pre-immune serum were employed as the negative control, while lysates from *B. duncani*-infected red blood cells reacted with rabbit polyclonal antibodies were utilized as the positive control. Finally, the membrane was immersed in peroxidase-conjugated goat anti-rabbit immunoglobulin G (diluted 1:5000) at room temperature for 1 h. These reactions were detected using Enhanced Chemiluminescence (ECL) detection from WesternBright^TM^ (Advansta, San Jose, CA, USA).

Indirect immunofluorescence assay (IFA) was employed to detect the intracellular localization of BdPyK I. Initially, slides containing infected *B. duncani* red blood cells were fixed with a cold solution composed of 10% acetone and 90% methanol at −20 °C for 30 min, alongside slides containing normal red blood cells. Subsequently, the slides were incubated with rabbit anti-rBdPyK I serum (diluted 1:200) and normal rabbit serum (as negative control, diluted 1:200) at 37 °C for 1 h. Following the incubation, the slides were washed three times with cold PBS solution and then incubated with Alexa-Fluor^®^ 488-conjugated goat anti-rabbit IgG secondary antibody (diluted 1:1000, Invitrogen) at 37 °C for 1 h. To stain the cell nuclei, the slides were further incubated with Hoechst dye (diluted 1:1000, Invitrogen). Additionally, an additional negative control was included, consisting of slides incubated only with the secondary antibody without the primary antibody incubation. Finally, the processed slides were imaged and observed under a fluorescence microscope.

### 2.5. Protein 3D Structure Modeling

To initiate the process of protein structure prediction, the first step was to obtain the amino acid sequence of the target protein. Subsequently, this sequence was submitted to AlphaFold for computational modeling (**https://alphafold.ebi.ac.uk/** (accessed on 5 January 2023)). Accessing the AlphaFold protein structure database or similar online services allowed retrieval of the execution code program, yielding predicted protein structures. Following AlphaFold’s completion of predicting the protein structure, subsequent structural and functional analyses could be conducted. The protein structure of HPyK I was retrieved from the PDB database (PDB: 1ZJH).

### 2.6. Ligand Preparation

A selection of commercially available small-molecule inhibitors (five types) were used as ligands in this study. Their 3D structures in SDF format were obtained from PubChem (https://pubchem.ncbi.nlm.nih.gov, accessed on 7 January 2023).

The compound IDs for the small molecules were as follows: PKM2 inhibitor (Compound CID: 131698387), shikonin (Compound CID: 479503), apigenin (Compound CID: 5280443), pioglitazone (Compound CID: 4829), and rosiglitazone (Compound CID: 77999). Discovery Studio 2018 Client (v18.1.100.18065) software, licensed to the State Key Laboratory of Agricultural Microbiology at Huazhong Agricultural University, Wuhan, Hubei, ensured a comprehensive optimization and docking strategy. Initially, Discovery Studio 3.5 employing the CHARMM force field was employed to remove all water molecules from the protein and add all hydrogen atoms. The binding site was defined as a sphere encompassing residues within 11.5 Å of the ligand, ensuring coverage of the natural ligand-binding region of the active site. CDOCKER (DS3.5) [30], Gold (Version 5.1) [30], and AutoDock Vina (Version 1.1.2) [31] were selected for virtual screening. Prior to docking the natural ligand to the active site of the protein structure, the scoring function and docking parameters underwent optimization.

### 2.7. Molecular Docking

The molecular docking of BdPyK I utilized Discovery Studio 2018 software version 18.1.100.18065. Structural preparation involved obtaining the crystal structure of BdPyK I from AlphaFold. Prior to docking, the structure model underwent optimization using Discovery Studio 2018 Client software version 18.1.100.18065. Optimization steps included removing water molecules and external atoms, detaching the original ligand, geometric refinement, adding hydrogen atoms, and identifying active sites. Docking protocol settings involved using the CDOCKER protocol in Discovery Studio. Key parameter configurations were as follows: number of poses generated (10), random conformation search (high), optimization direction (high), and simulated annealing enabled (true). All other parameters were set to default values.

### 2.8. Enzyme Kinetics and Inhibition Assays

This study aimed to investigate the effects of six small-molecule compounds on the enzyme activity of BdPyK I. These selected compounds, including PKM2 inhibitor, shikonin, tannic acid, apigenin, pioglitazone, and rosiglitazone, were initially dissolved in their respective stock concentrations using dimethyl sulfoxide (DMSO) or absolute ethanol as solvents. Subsequently, an indirect coupling lactate dehydrogenase assay was employed to evaluate the influence of these compounds on BdPyK I enzyme activity.

To conduct standard enzyme kinetics analysis, we employed a spectrophotometer (BioTek, located in Winooski, VT, USA) to monitor the degradation of NADH at 340 nm wavelength. At 37 °C, the oxidation of 1 micromole of NADH per minute was defined as one unit of PyK activity. Samples were measured continuously at 2, 7, 12, 17, 22, and 27 min. In order to investigate the influence of pH, Mg^2+^, and K^+^ concentrations on activity, tests were conducted within the pH range of 4.0–8.0 and Mg^2+^ and K^+^ concentrations ranging from 0 to 200 mM, including concentrations of 0, 25, 50, 75, 100, 125, 150, 175, and 200 mM. A reaction system with a total volume of 200 mL was designed, comprising 50 mM Tris-HCl buffer (pH 7.5), 50 mM MgCl_2_, 20 mM KCl, 3 mM PEP (MilliporeSigma), 3 mM ADP (MilliporeSigma), 0.5 mM NADH (MilliporeSigma), 100 ng recombinant lactate dehydrogenase (rBdLDH), and 100 ng BdPyK I. Enzyme activity was assessed by calculating the initial rate of NADH degradation within the first 5 min and determining the relative percentage activity. Additionally, under saturated conditions, it was found that 3 mM PEP and 3 mM ADP were the optimal reaction conditions, and the corresponding kinetic parameters of PEP and ADP were measured separately.

Under the conditions of 3 mM PEP and 3 mM ADP, the influence of six different small-molecule compounds on the enzymatic activity of BdPyK I was investigated across varying concentrations (0–500 μM). A control group was established to eliminate solvent interference on experimental results, ensuring that DMSO did not affect BdPyK I enzyme activity. The group with the addition of DMSO was designated as the negative control group. The inhibition rate was calculated using the following formula: Inhibitor (%) = (ΔAcontrol − ΔAsample)/ΔAcontrol × 100%. All experiments were independently repeated three times to enhance the reliability and accuracy of the results. Following completion of the experiments, enzyme activity data were analyzed using GraphPad Prism 8.0 software. IC_50_ values for each small-molecule compound were calculated using software analysis functions. The IC_50_ value represents the compound concentration required to decrease enzyme activity by 50% and serves as a fundamental parameter for evaluating the inhibitory effect of compounds on enzyme activity.

### 2.9. In Vitro Growth Inhibition Assay of Babesia duncani

Adjustments were made according to the solubility of compounds to prepare stock solutions with concentrations up to 15 mM. These stock solutions were subsequently diluted with culture medium or buffer to intermediate concentrations, serving as the basis for further concentration gradient dilutions. Concentration gradients were established, selecting concentrations ranging from 0.05 to 100 μM, approximating the IC_50_ range. The design of the concentration gradient was tailored for the experiment. To maintain an initial parasite rate of 1%, drug dilutions occupied 50% of the total volume in the 96-well plate, totaling 100 μL. It is important to note that drug concentrations should be twice the final concentration. The experiment comprised concentration gradient groups, blank groups, positive control groups, and DMSO groups. The DMSO group will serve as the reference group, while 5 nM WR99210 drug will be added as the positive control group, and the group without the addition of any drug will serve as the blank group [30]. Each group underwent three repetitions, with a 72 h incubation period. Data collection could be performed using a fluorescence assay. A total of 100 μL SYBR Green binding luminescent solution (Beyotime, Shanghai, China) was added, followed by incubation at 37 °C for 3 h. Fluorescence measurements were conducted using a multifunctional microplate reader, with excitation and absorption wavelengths set at 497 nm and 520 nm, respectively.

### 2.10. Enzyme Kinetics Experiments for PyK I Mutant Variants

The catalytic activity of purified BdPyK I and HPyK I, along with their 10 amino acid mutant variants (67A, 71A, 107A, 108A, 109A, 193A, 230A, 256A, 282A, 286A), was assessed using the purchased CheKine™ Pyruvate Kinase Activity Assay Kit (microplate method) from Abbkine (Wuhan, China). Purified proteins were added to the reaction at a concentration of 100 ng following the instructions provided in the assay kit manual. The experimental procedure was carried out according to the instructions provided in the assay kit manual. The OD difference in the reaction at 340 nm was determined using an enzyme-linked immunosorbent assay (ELISA) reader to ascertain the change in NADH at 20 s and 2 min 20 s after the start of the reaction. The difference in absorbance values between these two time points was calculated. The data are presented as mean ± standard deviation (SD), with n = 3. Enzyme activity data were analyzed using GraphPad Prism 8.0 software.

### 2.11. Statistical Analysis

The data were analyzed using GraphPad Prism 8 (San Diego, CA, USA) through two-way analysis of variance (ANOVA), followed by Tukey’s multiple comparison test. Results are presented as mean ± SD. *p* < 0.05 was considered significant difference. NS, no significance at the 5% level; *, *p* < 0.05 denotes significance at the 5% level; **, *p* < 0.01 signifies significance at the 1% level; and ***, *p* < 0.001 indicates significance at the 0.1% level. Error bars represent SD.

## 3. Results

### 3.1. Cloning and Evolutionary Analysis of the BdPyK I Gene

**Figure 1:**
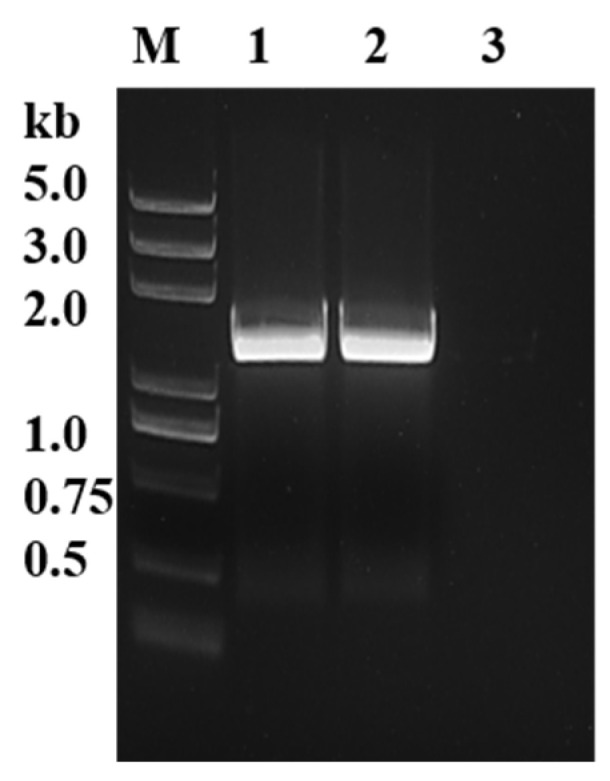
Identification of BdPyK gene through gDNA and cDNA amplification. M: DNA marker; 1: PyK I amplified by gDNA of *B. duncani*; 2: PyK I of *B. duncani* cDNA amplification; and 3: negative control (water).

The sequences of PyK I in *B. duncani* were initially aligned using NCBI database, and specific primers were then designed accordingly. Subsequently, the open reading frame (ORF) sequences of PyK I from both cDNA and gDNA of *B. duncani* were successfully amplified using PCR technology (Figure 1). The ORF sequences were 1542 bp in length, encoding 514 amino acids.

**Figure 2:**
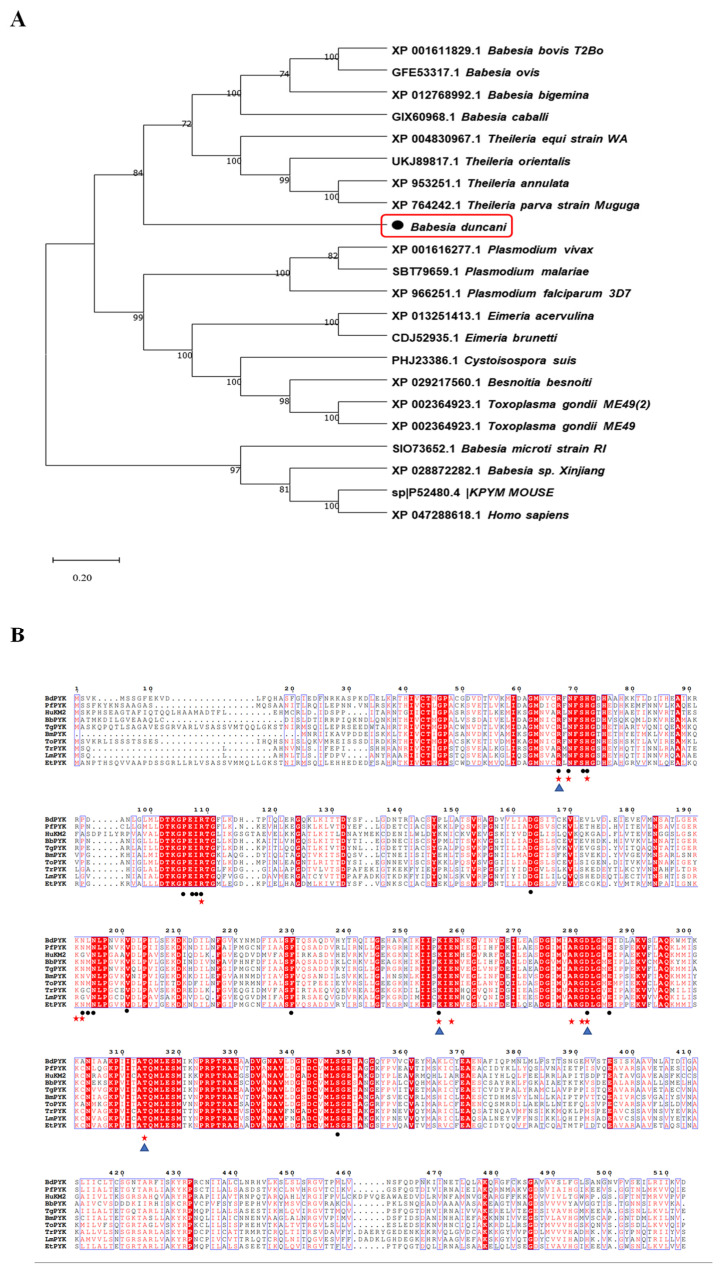
Phylogenetic tree analysis of BdPyK sequences in different species. (**A**) This figure illustrates the phylogenetic analysis based on the amino acid sequence of BdPyK. An adjacency tree was constructed to display the evolutionary relationships between BdPyK and other protozoan and mammalian PyKs. The scale bar represents the amino acid substitutions per site, while branch lengths reflect genetic distance variations between species. This analysis was performed using MEGA 11 software for data processing and visualization. Numbers and letters represent sequence identifiers in NCBI. (**B**) Amino acid sequence alignment of PyK I. Alignment results of amino acid sequences of PyK from *B. duncani*, Plasmodium falciparum, Homo sapiens, *B. bovis*, *T. gondii*, B. microti, *Theileria orientalis*, *Trypa-nosoma*, *Leishmania* spp, and *Eimeria tenella*. (• represent the active binding sites of BdPyK I, ★ represent the active binding sites of PfPyK I, and ▲ represent the active binding sites of HPyK I).

By multiple sequence alignment analysis, we identified amino acid sequences of PyK in *B. duncani* compared to those of other apicomplexan parasites (such as *Plasmodium*, *Theileria*, *Toxoplasma*, etc.) and mammalian PyK. The alignment results revealed that the amino acid sequence of PyK in *B. duncani* shares up to 80% sequence identity with those of apicomplexan parasites, including *Babesia bovis* (XP_001611829.1) and *Theileria annulata* (XP_004830967.1), indicating significant similarity of molecular structure between PyK of *B. duncani* and these apicomplexan parasites, as well as mammals (Figure 2A). Additionally, the amino acid sequence of PyK in *B. duncani* shares a homology of up to 42.69% with the amino acid sequence of PyK in Homo sapiens (XP_047288618.1, Figure 2A).

Due to the crucial role of amino acid residues in enzyme catalytic activity, substrate binding, or enzyme stability concerning small-molecule compounds, this study further analyzed the structural biology of the mechanism of action of small-molecule compounds on BdPyK I. A targeted mutagenesis strategy was employed to elucidate the roles of key amino acid residues in the interaction of small-molecule compounds. This strategy involved investigations of the PyK activity amino acid residues of various organisms, including *B. duncani*, *Plasmodium falciparum*, *Homo sapiens*, *B. bovis*, *T. gondii*, *B. microti*, *Theileria orientalis*, *Trypa-nosoma*, *Leishmania* spp, and *Eimeria tenella*. Additionally, the active binding sites of BdPyK I, PfPyK I, and HPyK I were analyzed and specially marked (Figure 2B).

### 3.2. Characteristics and Identification of the PyK I Gene

**Figure 3:**
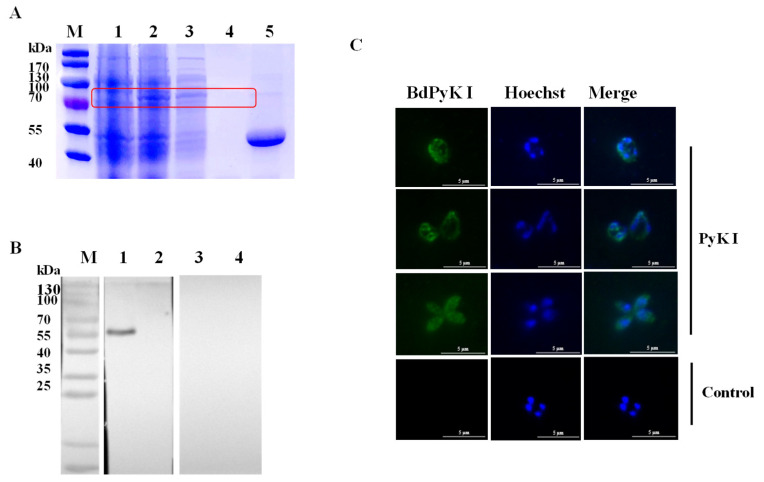
Purification and indirect immunofluorescence identification of recombinant BdPyK I protein. (**A**) Expression and purification identification of recombinant protein rBdPyK I. M: Protein molecular weight marker; 1: uninduced control; 2: induced expression; 3: soluble purified expression; 4: inclusion body expression; and 5: the concentrated BdPyK I protein after removal of the GST tag. The target bands are highlighted with red boxes for emphasis. (**B**) Identification of native BdPyK I. M: Protein marker; 1: lysates of *B. duncani* reacted with PyK I rabbit polyclonal antibody; 2: lysates of normal RBCs reacted with PyK I rabbit polyclonal antibody; 3: lysates of *B. duncani* reacted with serum from normal rabbit; and 4: lysates of normal RBCs reacted with serum from normal rabbit. (**C**) Localization of BdPyK I at single, double, and tetrad stages, respectively. Localization of the negative control in the intracellular phase. Anti-rBdPyK I serum (green) and Hoechst nuclear staining (blue). Scale bars: 5 μm.

The PyK I gene was amplified and subsequently cloned into the pGEX-6p-1 vector. The recombinant BdPyK I (rBdPyK I) protein with a fusion GST tag was expressed in *E. coli* BL21 (DE3) and purified to approximately 82 kDa (predicted as 83 kDa, Figure 3A). Subsequently, the GST tag of rBdPyK I was cleaved using the laboratory-preserved HRV 3C protease, yielding a 56 kDa product (Figure 3A). To authenticate natural BdPyK I, the lysate of *B. duncani* was incubated with rabbit polyclonal anti-BdPyK I serum and pre-immune rabbit serum. Western blotting analysis revealed a distinct 56 kDa band in the reaction between *B. duncani* lysate and anti-BdPyK I serum, consistent in size with the predicted BdPyK I. Additionally, no signals were detected with pre-immune serum and red blood cell lysate, serving as negative controls (Figure 3B). In order to better understand the characteristics of BdPyK I, the localization of BdPyK I in schizonts and infected red blood cells (iRBCs) was assessed using IFA, and BdPyK I was found within the cytoplasm of iRBCs (Figure 3C). No significant fluorescence was observed in the negative control group (Figure 3C).

### 3.3. Docking of Small-Molecule Compounds with PyK I Molecule

**Figure 4:**
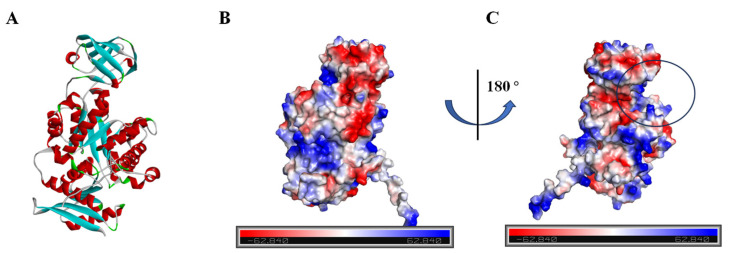
Analysis of the protein structure of BdPyK I predicted by AlphaFold. (**A**) The protein structure of BdPyK I predicted by AlphaFold, with α-helices represented in red, β-sheets in blue, and random coils (β-turns) in green. (**B**) Surface charge representation of the BdPyK I protein structure. Coloring according to electrostatic potential: regions with positive charges are depicted in blue, while regions with negative charges are shown in red. (**C**) After rotating 180° from (**B**), the active pocket (i.e., the binding site) of the BdPyK I structure becomes visible.

Given the absence of structural data for BdPyK I protein, protein structure prediction was conducted via AlphaFold software leveraging deep learning algorithms. The α-helices, β-strands, and irregular coils are pivotal constituents of protein structure, representing specific interactions and spatial arrangements of amino acid residues within the protein chain. Analysis of the electrostatic charge distribution on the surface of the BdPyK I protein structure holds significance for comprehending its intermolecular interactions. The electrostatic surface charge map illustrates positive charged regions depicted in blue and negative charged regions in red, with an electrostatic potential ranging from −62.840 kBT/e to 62.840 kBT/e. Assessment of the active pocket of BdPyK I revealed its potentiality to bind small-molecule compounds (Figure 4A–C).

**Figure 5:**
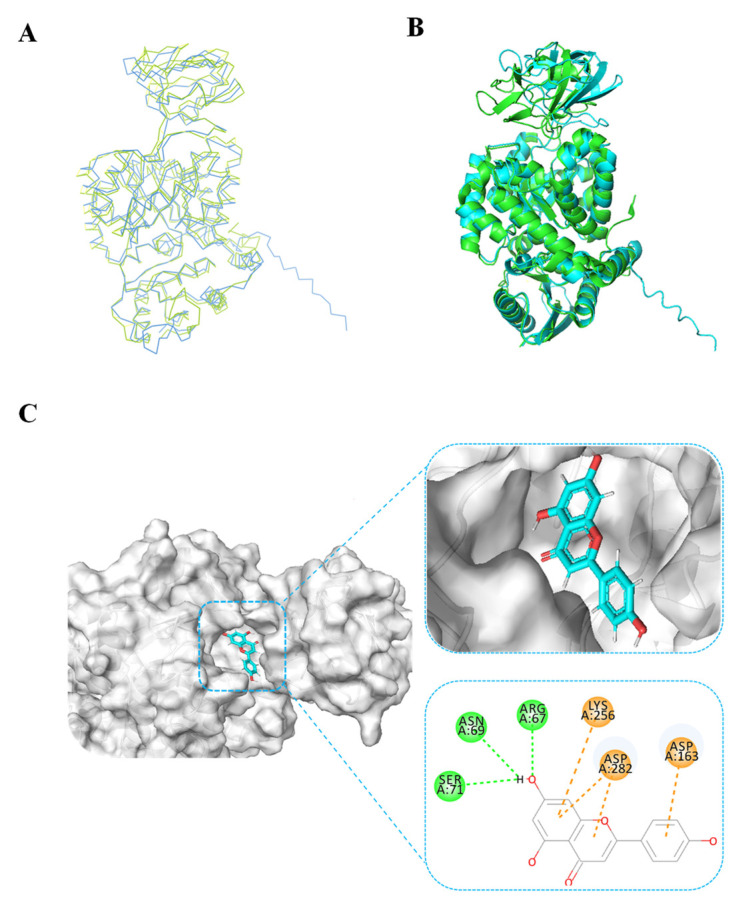
BdPyK I protein structure alignment and docking analysis of small-molecule compounds. (**A**) Linear sequence alignment between BdPyK I and HPyK (PDB: 1ZJH) proteins, with BdPyK I colored blue and HPyK green, revealing conserved regions and divergences. (**B**) Structural alignment of BdPyK I and HPyK proteins in their folded conformations, again with BdPyK I depicted in blue and HPyK in green, highlighting similarities and differences in their three-dimensional shapes and binding pockets. (**C**) Docking visualization of apigenin with BdPyK I demonstrates the chelation of apigenin within the binding pocket of BdPyK I. Key amino acid residues involved in the protein–ligand interaction are visualized.

**Figure 6:**
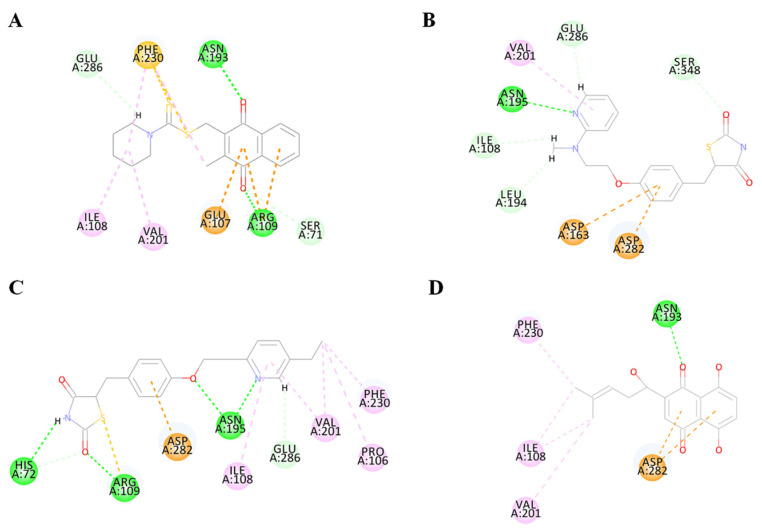
Diagram of the interaction between BdPyK I protein and small-molecule compounds. (**A**–**D**) Visualization of the amino acid sites involved in the interaction between BdPyK I and various compounds: (**A**) PKM2 inhibitor; (**B**) rosiglitazone; (**C**) pioglitazone; and (**D**) shikonin. These interactions reveal key binding interfaces and intermolecular forces that contribute to forming the protein–ligand complexes. Different amino acid colors correspond to different functional groups.

To validate the accuracy of the predicted structure, the BdPyK I protein was compared with a known similar protein, HPyK I (Figure 5A). The results demonstrated a substantial likeness, confirming the reliability of the predicted structure (Figure 5B). Subsequently, docking analysis was employed to investigate the interaction between small-molecule compounds and BdPyK I protein, disclosing specific binding of apigenin with the protein (Figure 5C). This study elucidates the structural characteristics of BdPyK I, providing crucial insights for pertinent drug design endeavors. Additionally, docking analysis of several small-molecule compounds, including shikonin (Figure 6D), pioglitazone (Figure 6C), rosiglitazone (Figure 6B), and PKM2 inhibitor (Figure 6A), was conducted using the same molecular docking method and followed by an analysis of their principal amino acid binding sites.

### 3.4. Enzyme Kinetics Study of BdPyK I

**Figure 7:**
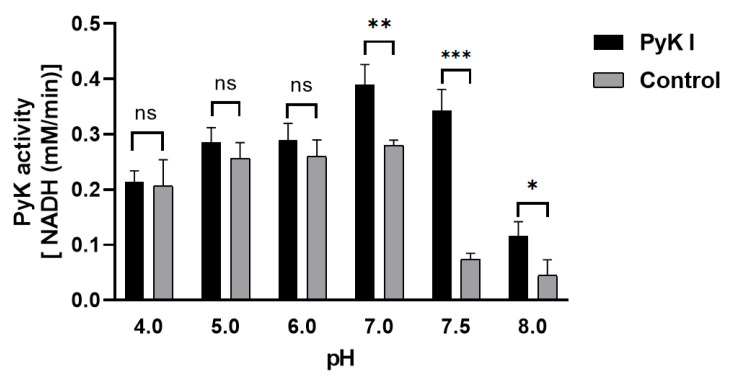
Changes in catalytic activity of BdPyK I under pH conditions. * *p* < 0.05, ** *p* < 0.01, *** *p* < 0.001, and ns, no significance. Error bars represent mean ± SD (*n* = 3), and all graphs were generated using GraphPad Prism 8.0.

Due to the inability to directly measure ATP and pyruvate levels, an indirect method was employed using coupled lactate dehydrogenase to determine PyK I catalytic activity by monitoring the decrease in NADH levels. This method aids in a more precise evaluation of BdPyK I enzyme activity. Optimal reaction conditions for BdPyK I enzyme kinetics experiments were preliminarily investigated. It was found that the reaction catalyzed by BdPyK I enzyme could proceed normally at a pH of 7.5, with a control group set up that did not contain BdPyK I as reference. Experimental results indicated that NADH might degrade over time under acidic conditions. Therefore, under neutral pH conditions (pH = 7.5), the enzyme reaction activity reached its optimum level (Figure 7).

**Figure 8:**
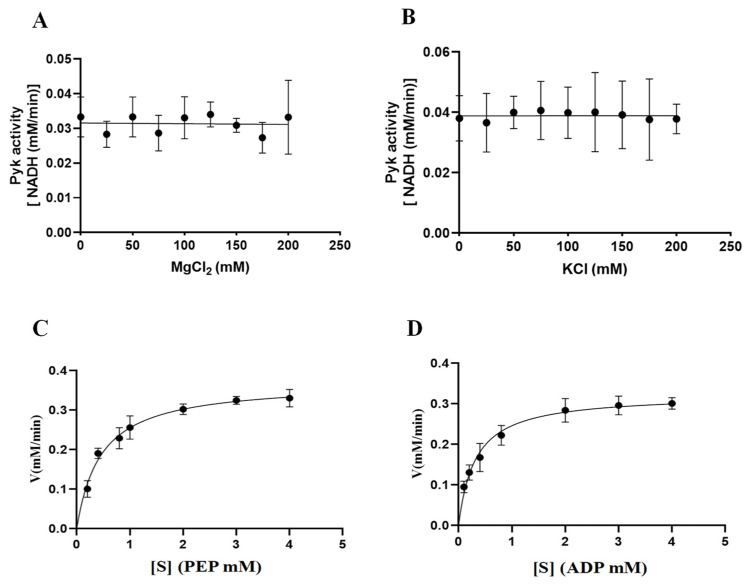
The catalytic activity of BdPyK I is influenced by the concentration gradients of Mg^2+^, K^+^, and the reactants. (**A**) Effects of gradient Mg^2+^ concentrations on the catalytic activity of BdPyK Ⅰ; (**B**) effects of gradient K^+^ concentrations on the catalytic activity of BdPyK Ⅰ; (**C**) the addition of a gradient concentration of PEP to PyK I catalyzes its activity; and (**D**) the addition of a gradient concentration of ADP to PyK I catalyzes its activity.

The involvement of cations (Mg^2+^ and K^+^) in the catalytic activity of PyKs in other apicomplexan parasites was established. This study extended this exploration to investigate the impact of varying concentrations of Mg^2+^ and K^+^ on the catalytic activity of BdPyK I, aiming to deepen insights into its catalytic mechanism. The results indicated that the catalytic activity of BdPyK I remained relatively stable with increasing concentrations of Mg^2+^ and K^+^ (Figure 8A–D). Subsequently, Michaelis–Menten equations for the reactants PEP and ADP were formulated based on gradient concentrations, and corresponding enzyme kinetics curves were delineated. The enzyme kinetics parameters of BdPyK I were also calculated (Figure 8A–D and Table 1), with KM values of 0.45 mM for PEP and 0.31 mM for ADP (Table 1). These findings established optimal conditions for enzyme kinetics experiments, with saturated reaction concentrations of 3 mM for both PEP and ADP. This study not only enhances our comprehension of the catalytic mechanism of BdPyK I but also provides important theoretical and experimental foundations for optimizing enzyme-catalyzed reaction conditions.

### 3.5. Inhibitor Screening of BdPyK I

**Figure 9:**
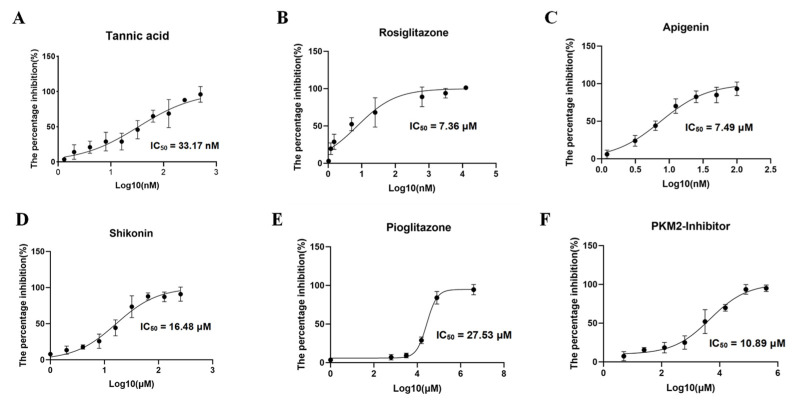
Inhibitor screening of BdPyK I. (**A**–**F**) These figures represent the inhibitory effects of six small-molecule compounds, including tannic acid, rosiglitazone, apigenin, shikonin, pioglitazone, and PKM2 inhibitor, on BdPyK I at various gradient concentrations. NADH absorbance (nmol) was measured at reaction times of 2, 7, 12, 17, 22, and 27 min, with background subtracted, at OD_340_. The data are presented as mean ± SD (*n* = 3).

Validation of the binding of six small-molecule compounds to the pocket of BdPyK I was undertaken by incorporating them into the catalytic reaction of BdPyK I at specific concentration gradients. Firstly, the overall enzyme activity of BdPyK I, coupled with BdLDH, was determined, and the individual effects of small-molecule compounds on BdLDH enzyme activity were separately evaluated to screen for potential compounds with inhibitory effects on BdPyK I. Test results revealed that PKM2 inhibitor, shikonin, apigenin, rosiglitazone, and pioglitazone effectively inhibited the catalytic activity of BdPyK I with minimal impact on BdLDH activity, indicating a specific inhibitory effect of these compounds on BdPyK I. Notably, at lower concentrations, tannic acid, apigenin, and rosiglitazone exhibited inhibitory effects on the catalytic activity of BdPyK I. While tannic acid and rosiglitazone also showed inhibitory effects on BdLDH activity, their inhibition on BdPyK I was more pronounced (Figure 9A–F). Therefore, tannic acid and rosiglitazone can also be considered as potential inhibitors of BdPyK I. In summary, by measuring enzyme reaction activity and screening small-molecule compounds, we successfully identified six potential drug molecules that significantly inhibit BdPyK I (Table 2).

### 3.6. Evaluation of Growth Inhibition Effect of Babesia duncani Cultured In Vitro

**Figure 10:**
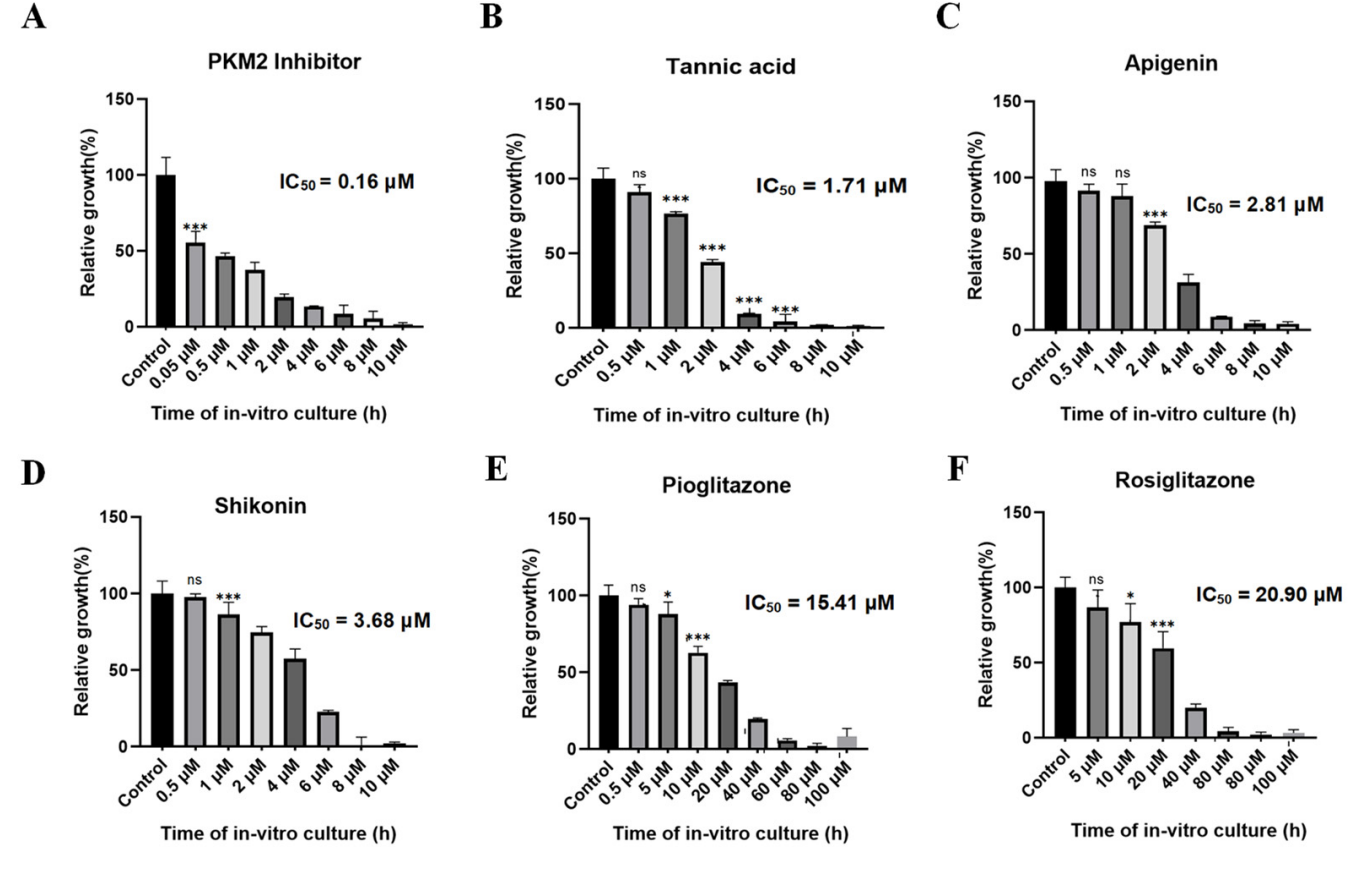
In vitro inhibitory effect of six small-molecule compounds on the growth of BdPyK I. (**A**–**F**) The in vitro growth inhibitory effects of six small-molecule compounds, including PKM2 inhibitor, tannic acid, apigenin, shikonin, pioglitazone, and rosiglitazone, on BdPyK I were regulated by gradient concentrations. * *p* < 0.05, *** *p* < 0.001, and ns, no significance. Error bars represent mean ± SD (*n* = 3), and all graphs were generated using GraphPad Prism 8.0.

Various concentrations of PKM2 inhibitor, shikonin, tannic acid, apigenin, pioglitazone, and rosiglitazone were introduced into a 96-well plate to assess their growth inhibitory effects on *B. duncani*. After 72 h of incubation, the parasite growth rate was determined using the SYBR Green method. Fluorescence enzyme activity was plotted using GraphPad Prism 8. Results unveiled a significant growth inhibition of PKM2 inhibitor at a concentration of approximately 2 μM, with an inhibition rate of 85% and an IC_50_ value of only 0.16 μM (Figure 10A). Tannic acid (Figure 10B), apigenin (Figure 10C), and shikonin (Figure 10D) also exhibited significant inhibitory effects on parasite growth, with IC_50_ values of 1.71 μM, 2.81 μM, and 3.68 μM, respectively. Additionally, pioglitazone (Figure 10E) and rosiglitazone (Figure 10F) inhibited parasite growth by 80% at concentrations of approximately 40 μM, with IC_50_ values of 15.41 μM and 20.9 μM, respectively.

The IC_50_ value represents a standard metric indicating the concentration of a drug or compound necessary to impede a biological process or inhibit cell growth. In this experiment, the IC_50_ values of six different compounds against *B. duncani* in vitro were compared with their IC_50_ values for BdPyK I activity. It was observed that the PKM2 inhibitor, apigenin, tannic acid, and shikonin showed significant potentialities as inhibitors of BdPyK I (Table 3). To contextualize these findings, the CC_50_ values for vero cells and HFF cells from cytotoxicity assay data [22] were considered collectively. Remarkably, the PKM2 inhibitor demonstrated remarkably high in vitro activity while exhibiting relatively low toxicity to both vero cells and HFF cells (Table 3). Tannic acid and apigenin also displayed notable in vitro activity with comparatively low toxicity toward these cell lines. However, shikonin exhibited relatively reduced in vitro activity against *B. duncani*, albeit showing higher toxicity to HFF cells (Table 3). Considering the activity, selectivity, and potential toxicity, this study conducted a comprehensive evaluation of the selectivity index (SI). It was found that the PKM2 inhibitor, tannic acid, and apigenin exhibit relatively high selectivity index values, making them promising characteristic inhibitors targeting BdPyK I.

### 3.7. Analysis and Validation of the Active Sites of BdPyK I Amino Acids

Based on the alignment of active amino acid sequences of PyK from multiple species (Figure 2B) and the binding sites of small-molecule compounds (Figure 6), ten key amino acid residues relevant to the binding of BdPyK I were selected. These residues are Arg67, Ser71, Glu107, Ile108, Arg109, Asn193, Phe230, Lys256, Asp282, and Glu286 (Table 4). Amino acid side chains play crucial roles in protein functionality, with their hydrophobic, hydrophilic, acidic, and basic properties intricately influencing interactions between amino acids, thereby determining protein structure and function. Different functional groups in amino acid side chains exert varying effects within proteins. For instance, the side chain of alanine contains only a methyl group (-CH₃), resulting in minimal spatial hindrance to protein structure (Table 4). Consequently, substituting alanine for an amino acid typically preserves the overall conformation of the protein, thereby aiding in maintaining its functionality and stability.

**Figure 11:**
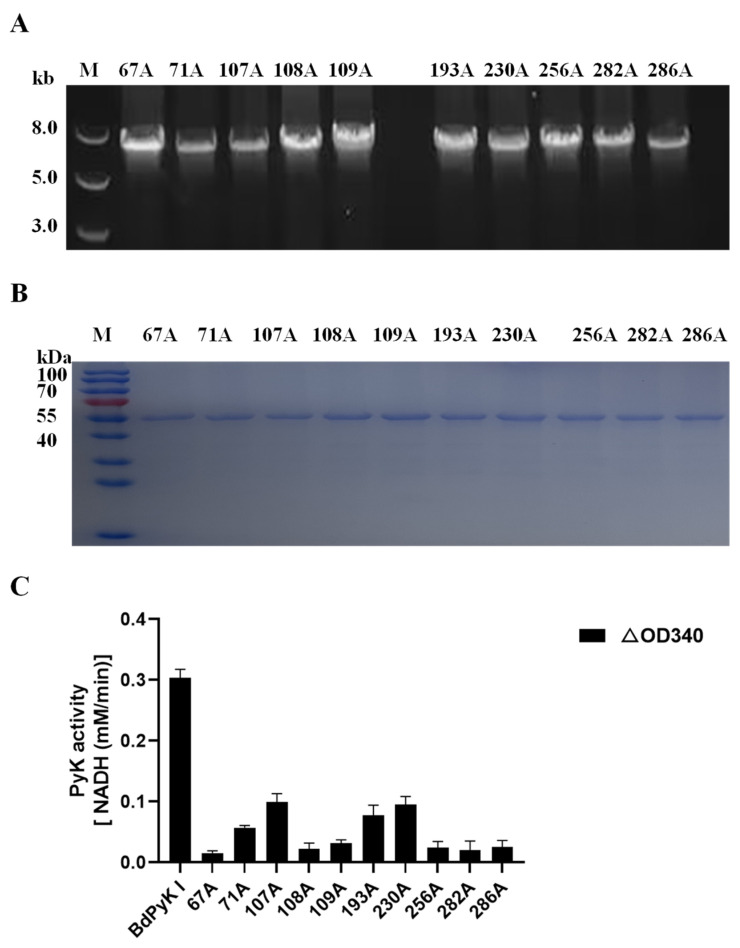
Sequence amplification of the mutant amino acid BdPyK I. (**A**) Amplification of the BdPyK I gene. (**B**) Purification and expression identification of BdPyK I protein. (**C**) Identification of the catalytic activity of BdPyK I. Using a spectrophotometer, the absorbance values at 340 nm were measured to determine the reduction in NADH at 20 s and 2 min 20 s after the start of the reaction. The difference in absorbance values between these two time points was calculated. The data are presented as mean ± standard deviation (SD), with *n* = 3.

The 10 amino acid residues within BdPyK I were mutated to alanine, and the effects of these mutations on catalysis were explored by expressing the corresponding mutant enzymes. The mutant fragments were successfully amplified using the wild-type pGEX-6p-BdPyK I plasmid and expressed in *E. coli* BL21 cells (Figure 11A,B). Subsequently, mutant proteins were purified by GST affinity chromatography and removal of the GST tag. The purified mutant proteins displayed sizes consistent with wild-type BdPyK I, indicating that the mutations did not alter the protein’s molecular weight (Figure 11B). The catalytic activity of the mutant BdPyK I was then assessed using the CheKine™ PK activity assay kit. The results showed that the enzymatic activity of almost all mutants significantly decreased (Figure 11C), suggesting that these amino acids are crucial for maintaining the structure and function of BdPyK I. These amino acids may directly affect substrate binding and catalytic activity or regulate catalytic efficiency by influencing enzyme stability and subunit interactions.

### 3.8. Isoleucine and Phenylalanine Are Critical Amino Acid Residues of HPyK I 

**Figure 12:**
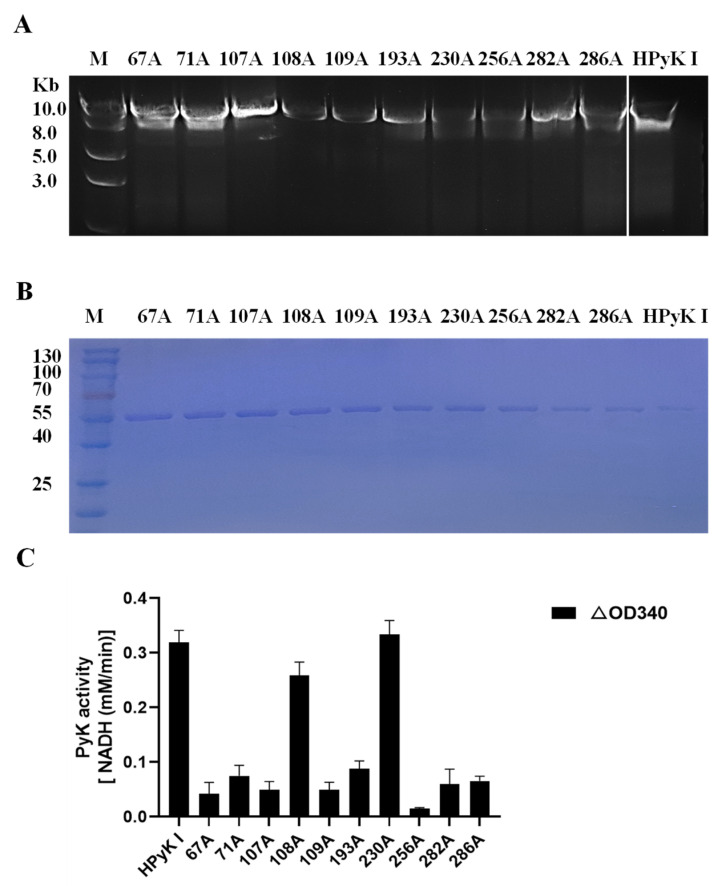
Sequence amplification of the mutant amino acid HPyK. (**A**) Amplification of the HPyK I gene. (**B**) Purification and expression identification of HPyK I protein. (**C**) Identification of the catalytic activity of HPyK I. Using a spectrophotometer, the absorbance values at 340 nm were measured to determine the reduction in NADH at 20 s and 2 min 20 s after the start of the reaction. The difference in absorbance values between these two time points was calculated. The data are presented as mean ± standard deviation (SD), with *n* = 3.

To investigate the impact of specific amino acid residues on the catalytic activity of HPyK I, a series of experimental procedures were conducted in this study. Firstly, wild-type pGEX-6p-HPyK I and mutation fragments targeting specific amino acid residues (67A, 71A, 107A, 108A, 109A, 193A, 230A, 256A, 282A, 286A) were successfully amplified (Figure 12C). Subsequently, validated mutant plasmids were transformed into *E. coli* BL21 cells, and the wild-type and mutant HPyK I recombinant proteins were expressed using an efficient *E. coli* expression system. Following induction and purification, wild-type HPyK I protein and 10 mutant HPyK I proteins were obtained from *E. coli* BL21 cells (Figure 12B). The experimental results demonstrated successful purification of wild-type HPyK I protein and 10 mutant HPyK I proteins, with the mutant variants exhibiting consistent protein molecular weights with the wild-type, laying the foundation for subsequent catalytic activity analysis.

The catalytic activity of mutant HPyK I proteins was assessed using the CheKine™ Pyruvate Kinase Activity Assay Kit (microplate method). The experimental findings revealed that compared to wild-type HPyK I, the enzymatic activity of most mutant amino acids decreased by over 85% (Figure 12C). This underscores the critical roles these amino acids play in maintaining the normal catalytic function of HPyK I. Notably, while most mutations led to a significant decrease in enzyme activity, mutants 108A (isoleucine) and 230A (phenylalanine) retained partial levels of catalytic activity. This observation implies that the roles these two amino acids played in the enzyme’s structure or function may differ from those of other mutated amino acids or they may partially retain catalytic capability through certain mechanisms. Based on these findings, we suspect that these amino acids may be directly involved in substrate binding and catalysis or influencing enzyme catalytic efficiency through stability and subunit interactions. These results are expected to offer novel insights for understanding the catalytic mechanism of HPyK I and for developing related therapeutics.

## 4. Discussion

This study focused on BdPyK I and identified tannic acid and apigenin, known inhibitors of PKM2, as specific inhibitors of PyK I, showing potentiality as lead compounds for development. Enzymatic activity assays of key active amino acid mutants revealed significant differences in the active pockets of BdPyK I and HPyK I, particularly at the critical amino acid sites isoleucine and phenylalanine. These findings provide important insights into the functional differences between BdPyK I and HPyK I.

The analysis of predicted structural features of the BdPyK I protein aims to elucidate the interaction between its active pocket and small molecular compounds, a critical step in understanding its function and biological significance. Molecular docking techniques were employed to align the predicted protein structure with five small molecular compounds, elucidating their binding modes and interactions within the active pocket. The active pocket is crucial for BdPyK I function, where its inherent shape and chemical properties are essential for recognizing and binding specific substrates or inhibitors. The binding of small molecular compounds to the active pocket is typically facilitated through hydrogen bonds, ionic bonds, van der Waals forces, or hydrophobic interactions [31,32,33]. Amino acid residues at the active site exhibit specificity to different small molecular compounds. Exploration of these differences enables us to identify compounds with potential biological activity. This process facilitates the screening of compounds for further research or drug design, providing candidate molecules for investigation.

Enzymatic characterization of BdPyK I validated the docking results. NADH’s absorbance values continuously decreased in both the control and experimental groups under slightly acidic conditions. This decrease occurs because, in acidic environments, the reduced form of NADH is susceptible to losing electrons, leading to its decomposition and release of hydrogen ions (H^+^), converting it to NAD^+^ (the oxidized form of nicotinamide adenine dinucleotide), which directly affects the absorbance of NADH. Therefore, within the pH range of 4.0–8.0, neutral conditions at pH 7.5 were determined to be optimal for BdPyK I enzyme activity.

Mg^2+^ and K^+^ play a crucial role in the binding and coordination process of the substrate. During the catalysis of mammalian PyK, the active site not only interacts with the two substrates PEP and ADP, where ADP forms a complex with Mg^2+^, but is also occupied by the monovalent cation K^+^ and the additional enzyme-bound divalent cation Mg^2+^. These interactions promote enzymatic reactions. Specifically, one of the catalytic lysine residues stabilizes the five-coordinated transition state generated during the direct transfer of phosphate from PEP to ADP, ensuring the smooth progression of the reaction [34]. However, adjustment of Mg^2+^ and K^+^ concentrations did not affect BdPyK I enzyme activity, suggesting that Mg^2+^ and K^+^ may not directly participate in the catalytic process. The catalytic mechanism of BdPyK I appears not to rely on Mg^2+^ and K^+^, thereby fluctuations in their concentrations do not significantly impact BdPyK I activity. By determining reaction rates at different substrate concentrations, the enzyme’s kinetic constants (Michaelis–Menten constant and maximum reaction rate) were obtained, reflecting the affinity between the enzyme and substrates as well as the catalytic efficiency of the enzyme. Refining the kinetic parameters of BdPyK I enzyme is crucial as it provides a more accurate and reliable basis for subsequent inhibitor screening experiments.

The PKM2 inhibitor demonstrated the highest selectivity index, representing a promising discovery. As a novel naphthoquinone derivative based on shikonin modification, the PKM2 inhibitor exhibits robust inhibitory capabilities against the tumor cell-specific M2 subtype of PyK and shows significantly enhanced inhibition compared to its precursor, shikonin [35]. Shikonin, a naphthoquinone natural pigment extracted from plants, possesses a unique chemical structure and biological activities. Naphthoquinone derivatives with specific biological activities or application properties can be synthesized by modifying shikonin, thus expanding its application scope in the pharmaceutical field [36]. Based on shikonin modification, naphthoquinone derivatives hold promise for inheriting or enhancing various biological activities such as anti-tumor, anti-inflammatory, antiviral, and antiparasitic effects, providing strong support for the development of novel drugs. The PKM2 inhibitor, as a novel naphthoquinone derivative based on shikonin modification, exhibits more potentialities than shikonin in anticancer drug development. PKM2 inhibitor locks PKM2 in a low-activity conformation, inducing metabolic changes in cancer cells, further highlighting its significant value in anticancer drug development [26,35]. The significant inhibitory effect of apigenin on BdPyK I enzyme activity is important in studying the interaction between apigenin and Babesia parasites. Despite the high toxicity of apigenin observed in in vivo experiments with Babesia rodents, its inhibitory effect on BdPyK I enzyme activity suggests that it may disrupt the metabolic processes of the Babesia parasites [22]. However, further in vivo experiments are needed to evaluate the toxicity of apigenin against *B. duncani* and consider factors such as the metabolism and distribution of apigenin in vivo. Given the potential high toxicity of apigenin, its chemical structure and biological activity can be adjusted through chemical modification, structural modification, combination with other compounds, and utilization of nanotechnology and biotechnology. By delving into apigenin’s bioactivity mechanism, toxicity mechanism, and structure–activity relationship, theoretical support and guidance can be provided for developing safer and more effective analogs or derivatives [37,38]. These modified compounds may have broader application prospects, offering new drug candidates for treating various diseases in the pharmaceutical field.

Structural analysis of BdPyK I and HPyK I, alongside site-directed mutagenesis, elucidated the essential role of key amino acid residues in enzymatic activity. While all mutant variants of BdPyK I lost enzymatic activity, the 108A (isoleucine) and 230A (phenylalanine) mutants of HPyK I retained catalytic functions. Moreover, competitive inhibition of the catalytic pocket of triosephosphate isomerase (TPI) by phosphoenolpyruvate (PEP) represents an intricate regulatory mechanism influencing the pentose phosphate pathway (PPP). Substitution of the isoleucine residue located within the TPI catalytic pocket with valine or threonine alters the binding mode of substrates and PEP, leading to decreased TPI activity both in vitro and in vivo [39]. This discovery highlights the importance of isoleucine in the catalytic process of TPI and provides new strategies for regulating PPP activity. Furthermore, transgenic yeast cells with these TPI mutations exhibited an accumulation of PPP intermediates and changes in stress resistance, simulating the activation of the PK-TPI feedback loop, further validating the critical role of isoleucine in metabolic regulation. Therefore, based on this research, it is reasonable to speculate that isoleucine may exhibit differences in PyK I across different species. These differences may arise from evolutionary divergence between species, varying metabolic demands, or other biological differences. In conclusion, the variation in isoleucine in PyK I provides a new perspective for understanding and regulating metabolic processes.

Phenylalanine plays a crucial role in the PyK I function, with studies highlighting its competitive binding with substrates ADP and PEP, thereby inhibiting cerebral PyK (an isozyme of PyK) activity and implicating its crucial role in metabolic regulation. Furthermore, phenylalanine, phenylpyruvate, and alanine act on a common site of cerebral PyK, which may be an allosteric site of the enzyme [40]. Allosteric sites are regions on enzyme molecules capable of binding regulatory molecules and altering enzyme activity. By binding to these amino acids, the activity of PyK can be modulated, thereby impacting glucose metabolism. For individuals with phenylketonuria, inhibition of cerebral PyK activity may be associated with reduced glucose metabolism in the brain. As PyK is a key enzyme in the glycolytic pathway, a decrease in its activity may hinder glucose metabolism, thereby affecting the energy supply and function of neuronal cells. This could be a mechanism underlying neurological dysfunction in patients with phenylketonuria. Therefore, the role of phenylalanine in PyK I is not only direct regulation of enzyme activity but also potentially closely related to the pathogenesis of metabolic diseases.

In this study, PKM2 inhibitor, apigenin, and apigenin were screened as potential specific inhibitors. A comprehensive analysis of the effects of differential amino acids (isoleucine and phenylalanine) in the active pockets of BdPyK I and HPyK I was conducted. Our findings provide a robust theoretical basis for the development of novel and efficient PyK I lead compounds. Through an in-depth exploration of key amino acids in the PyK I active pocket, the interaction mechanism between inhibitors and enzymes can be better understood, and complex regulatory networks potentially involved in metabolic diseases can be revealed. These findings offer novel strategies for the prevention and treatment of metabolic diseases.

## 5. Conclusions

This study confirmed that PKM2 inhibitor, apigenin, and apigenin are specific inhibitors of PyK I, demonstrating significant potentiality as lead compounds for further development. Furthermore, we discovered that isoleucine and phenylalanine exhibit significant differences in the active pockets of BdPyK I and HPyK I through the enzymatic activity measurement of key active amino acid mutants. These two crucial amino acid residues provide important clues for understanding the functional differences between the two enzymes. These findings deepen our understanding of PyK I action mechanism and provide a valuable theoretical basis for subsequent drug design and development.

## Figures and Tables

**Table 1:** The impact of gradient concentrations of PEP and ADP on the kinetic parameters of BdPyK I.

Parameters	PEP	ADP
*K*_M_ (mM)	0.45 ± 0.104	0.3136 ± 0.024
*V*_max_ (mM/min)	0.3701 ± 0.015	0.3234 ± 0.018
*K*_cat_ (S^−1^)	18.3	16
*K*_cat_/*K*_M_ (S^−1^ · M^−1^)	4.01 × 10^4^	5.10 × 10^4^

**Table 2:** Summary table of inhibitor screening for BdPyK I.

Compounds	IC_50_ (μM)	
	PyK I (μM)	LDH (μM)
Tannic acid	0.03	1.32
PKM2 inhibitor	10.89	-
Apigenin	7.49	-
Shikonin	16.48	-
Pioglitazone	27.53	-
Rosiglitazone	7.36	1.35 × 10^5^

**Table 3:** Evaluation of inhibitor selectivity index.

	IC_50_ (μM)		CC_50_ (μM)		Selectivity Index (SI)	
Name	PyK I Activity	*B. duncani*	Vero	HFF	Vero	HFF
Tannic acid	0.03	1.71 ± 0.33	53 ± 2.37	36.46 ± 3.88	31	21
PKM2 inhibitor	10.89	0.16 ± 0.08	39.27 ± 8.32	18.66 ± 1.99	243	116
Apigenin	7.49	2.81 ± 1.06	58.23 ± 2.67	35.99 ± 2.95	20.64	12.8
Shikonin	16.48	3.68 ± 0.48	2.34 ± 0.29	0.63 ± 0.32	0.64	0.17

**Table 4:** Summary of amino acid mutations.

Amino Acid Residue Numbers	Mutated Amino Acids
67A	ARG
71A	SER
107A	GLU
108A	ILE
109A	ARG
193A	ASN
230A	PHE
256A	LYS
282A	ASP
286A	GLU

## Data Availability

The original contributions presented in the study are included in the article/Supplementary Materials, further inquiries can be directed to the corresponding author.

## Supplementary Materials

The following supporting information can be downloaded at: https://www.mdpi.com/article/10.3390/microorganisms12061141/s1, Table S1: About the primers related to gene amplification.
